# Potential genes and pathways associated with heterotopic ossification derived from analyses of gene expression profiles

**DOI:** 10.1186/s13018-021-02658-1

**Published:** 2021-08-14

**Authors:** Zhanyu Yang, Delong Liu, Rui Guan, Xin Li, Yiwei Wang, Bin Sheng

**Affiliations:** 1 Department of Orthopaedics and Traumatology, Hunan Provincial People’s Hospital, the First Affiliated Hospital of Hunan Normal University, No. 61 Jiefang West Road, Changsha, Hunan 410000 People’s Republic of China; 2 Hunan Emergency Center, No. 90 Pingchuan Road, Changsha, Hunan 410000 People’s Republic of China

**Keywords:** Heterotopic ossification, Bioinformatic analysis, Differentially expressed genes, Protein–protein interaction, Functional enrichment analysis

## Abstract

**Background:**

Heterotopic ossification (HO) represents pathological lesions that refer to the development of heterotopic bone in extraskeletal tissues around joints. This study investigates the genetic characteristics of bone marrow mesenchymal stem cells (BMSCs) from HO tissues and explores the potential pathways involved in this ailment.

**Methods:**

Gene expression profiles (GSE94683) were obtained from the Gene Expression Omnibus (GEO), including 9 normal specimens and 7 HO specimens, and differentially expressed genes (DEGs) were identified. Then, protein–protein interaction (PPI) networks and Gene Ontology (GO) and Kyoto Encyclopedia of Genes and Genomes (KEGG) enrichment analyses were performed for further analysis.

**Results:**

In total, 275 DEGs were differentially expressed, of which 153 were upregulated and 122 were downregulated. In the biological process (BP) category, the majority of DEGs, including EFNB3, UNC5C, TMEFF2, PTH2, KIT, FGF13, and WISP3, were intensively enriched in aspects of cell signal transmission, including axon guidance, negative regulation of cell migration, peptidyl-tyrosine phosphorylation, and cell-cell signaling. Moreover, KEGG analysis indicated that the majority of DEGs, including EFNB3, UNC5C, FGF13, MAPK10, DDIT3, KIT, COL4A4, and DKK2, were primarily involved in the mitogen-activated protein kinase (MAPK) signaling pathway, Ras signaling pathway, phosphatidylinositol-3-kinase/protein kinase B (PI3K/Akt) signaling pathway, and Wnt signaling pathway. Ten hub genes were identified, including CX3CL1, CXCL1, ADAMTS3, ADAMTS16, ADAMTSL2, ADAMTSL3, ADAMTSL5, PENK, GPR18, and CALB2.

**Conclusions:**

This study presented novel insight into the pathogenesis of HO. Ten hub genes and most of the DEGs intensively involved in enrichment analyses may be new candidate targets for the prevention and treatment of HO in the future.

## Background

Heterotopic ossification (HO) represents pathological lesions, referred to as the development of heterotopic bone in extraskeletal tissues around joints, which often occurs in the elbow, thigh, pelvis, and shoulder [1, 2]. The clinical manifestations were altered with the progression of the disease. Local pain, tenderness, swelling, and stiffness of the involved joints were present in the early stage of HO. At the end of the disease, patients even present with complete ankyloses, which seriously damages their quality of life [3]. The specific causes of HO were still uncertain. It is generally believed that the occurrence of HO is related to genetic susceptibility and severe trauma. Progressive ossifying fibrous dysplasia (FOP), a genetic hereditary form of HO, is extremely rare, with a prevalence of approximately 1:2,000,000 in the population [4–7]. However, there was a much higher incidence of HO following soft-tissue trauma, amputations, and central nervous system injury, such as cerebral anoxia, encephalitis, traumatic brain injuries, and spinal cord lesions [8, 9].

At present, the only effective way to treat HO is surgical resection of ossified tissue. Since HO is prone to recurrence, this method is only temporarily effective [10]. Moreover, when the ossification tissue invades the large blood vessels and nerves, the complication rate caused by resection is also increased. Studies have shown that HO is a disease with unknown pathogenesis, inadequate prevention and treatment, and a high disability rate [11]. Therefore, to find a more effective therapeutic method, we need to better understand the pathogenesis of HO [12].

Recently, translational medicine has provided a new way of thinking that connects basic medical research with clinical treatment [13]. Bioinformatics based on genomic genetics and omics chips has a close interaction with systems medicine theory and automatic communication technology, which speeds up the industrialization process of the transformation from scientific research to clinical application. Its application in orthopedics will also lead to the rapid shortening of the distance between foundation and clinic and a new generation of orthopedic surgeons [14]. The bioinformatics analysis of gene expression, which serves as a key technology in mechanistic exploration, plays an important role in screening gene mutations and studying genetic behavior.

Our study screened out the genes differentially expressed in bone marrow mesenchymal stem cells (BMSCs) from HO, which were identified from a public dataset. In this work, we sought to synthesize protein–protein interaction (PPI) network analysis and Gene Ontology (GO) and Kyoto Encyclopedia of Genes and Genomes (KEGG) enrichment analysis among DEGs to explore the potential mechanism of HO and candidate gene targets, which may be used to prevent, alleviate and even reverse the progression of HO.

## Methods

### Microarray data resource

A gene expression profile dataset (GSE94683), GSE94683 [15], a gene expression profile dataset obtained from a public genomics data repository (GEO database, http://www.ncbi.nlm.nih.gov/geo/), was produced on the GPL10630 Agilent-021531 Whole Human Genome Oligo Microarray 4x44K. According to the annotation information in the platform, the probes were alternated into corresponding gene symbols. GSE94683 contains 9 normal specimens and 7 HO specimens. HO specimens were obtained from patients with injuries at Garches Hospital, Garches, France. Healthy specimens were obtained from patients after total hip surgery at Blois Hospital (Blois, France) and at Centre de Transfusion Sanguine des Armées (Percy Hospital, Clamart, France).

### Data processing and identification of DEGs

Principal components analysis (PCA) and normalization were performed by ggbiplot and the preprocessCore package in R. After the preprocessing of the datasets, the identification of DEGs was processed using multiple linear regression models via the limma package of R language. A probe without any corresponding gene symbol was eliminated, and a gene with many probes was averaged. All DEGs and the top 30 DEGs were demonstrated via a volcano plot, which was performed by the ggplot2 package. DEGs were screened by using classical *t* test and |Log2(FoldChange)| > 2 and adj. *P* < 0.05 was defined as a threshold and criteria for the identification of statistically significant DEGs. The relative expression values of total DEGs and the top 30 upregulated and downregulated DEGs, which were extracted from the gene expression profile, were demonstrated by the hierarchical clustering heatmaps via the pheatmap package of R language.

### Functional enrichment analysis for DEGs

KEGG is a data reservoir for unravelling advanced biological functions and pathways involved with genomic information [16]. GO is a tool for defining concepts or classifying gene biological functions by analyzing a large list of genes [17]. The Database for Annotation, Visualization and Integrated Discovery (DAVID), a web-based database resource that explores gene data, facilitates researchers to reveal potential genetic significance [18]. DAVID was used to perform the GO and KEGG enrichment analyses. The functional enrichment analyses were processed via the ggplot2 package in R and demonstrated by bubble plots.

### Integration of PPI network analysis

The Search Tool for the Retrieval of Interacting Genes (STRING), an original online program that predicts the relationships and interactions among proteins involved [19], was applied to construct a functional network among proteins. Cytoscape, an open-access visual tool, was able to visualize processes and interactions among proteins. Molecular Complex Detection (MCODE), a plugin of Cytoscape, was used to explore intensive communications and discover the most significant module. “MCODE scores ≥ 5”, “k-score = 2”, “Max depth = 100”, “node score cut-off = 0.2,” and “degree cut-off = 2” were selected as criteria for the identification of significant modules.

### Hub genes selection and analyses

CytoHubba, a plugin of Cytoscape applied with 12 topological analysis methods, was used to detect more comprehensive hub genes and avoid missing data. According to MCC, MNC, DMNC, and Degree, the top 25 genes were predicted. Venn diagrams were used to screen the hub genes by identifying the overlapping genes.

## Results

### Data source and data preprocessing

**Fig. 1:**
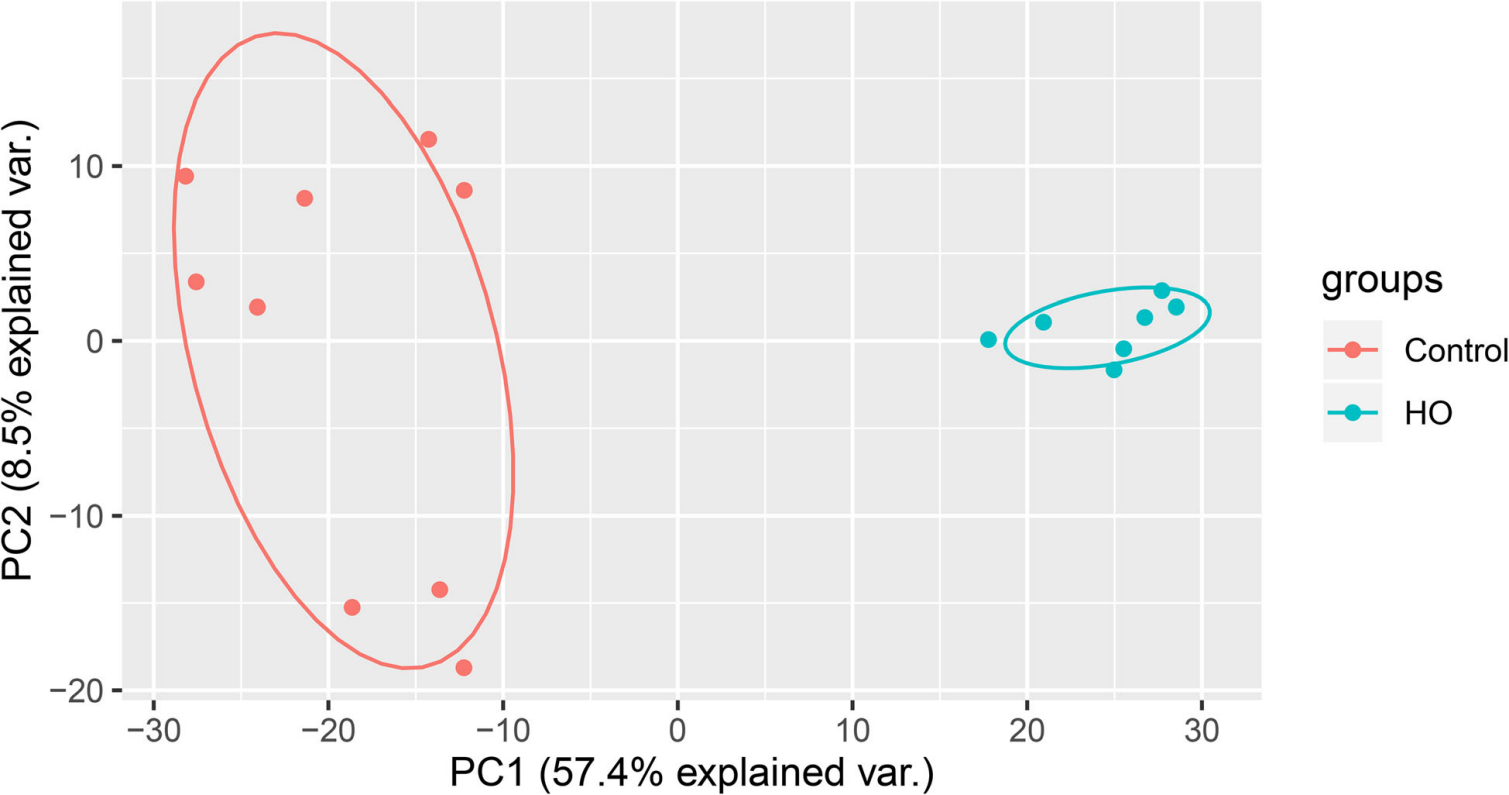
Principal components analysis of datasets. Red represents samples from normal cohort. Blue represents samples from heterotopic ossification

**Fig. 2:**
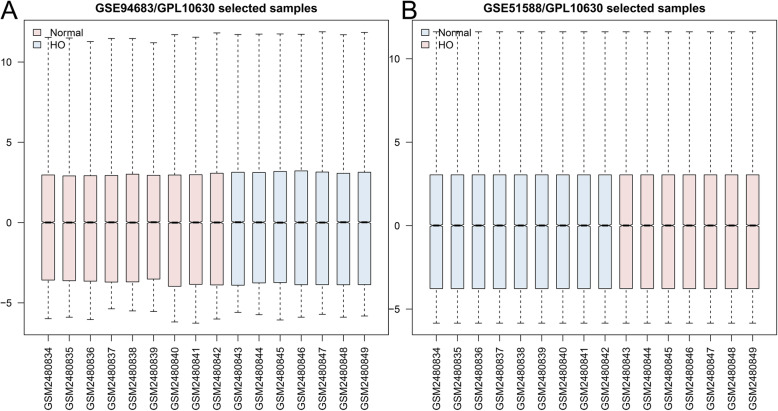
Normalization of the sample data demonstrated by box plots. **A** Before processing and **B** after processing. HO = heterotopic ossification, DEGs = differentially expressed genes

As large amounts of data were integrated into a gene expression profile (GSE94683), the original data were downloaded from the GEO database. PCA of datasets demonstrated that there were significant differences between HO samples and control samples (Fig. 1). Normalization of the data is presented in a boxplot (Fig. 2).

### DEGs in HO samples compared with control samples

**Fig. 3:**
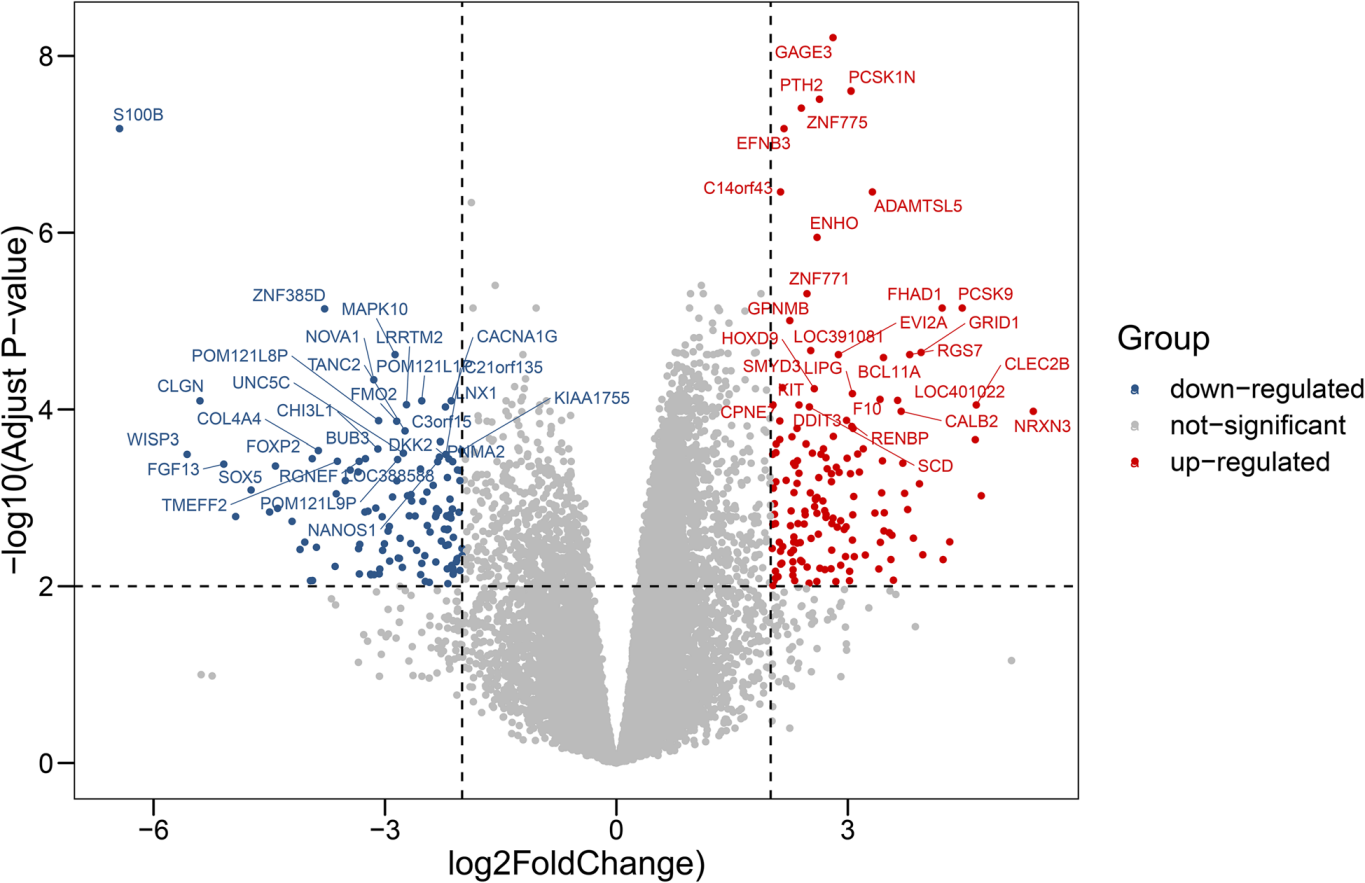
DEGs screening identified by volcano plot. Grey refers to no difference in expression. Red refers to up-regulated expression. Blue refers to down-regulated expression

**Fig. 4:**
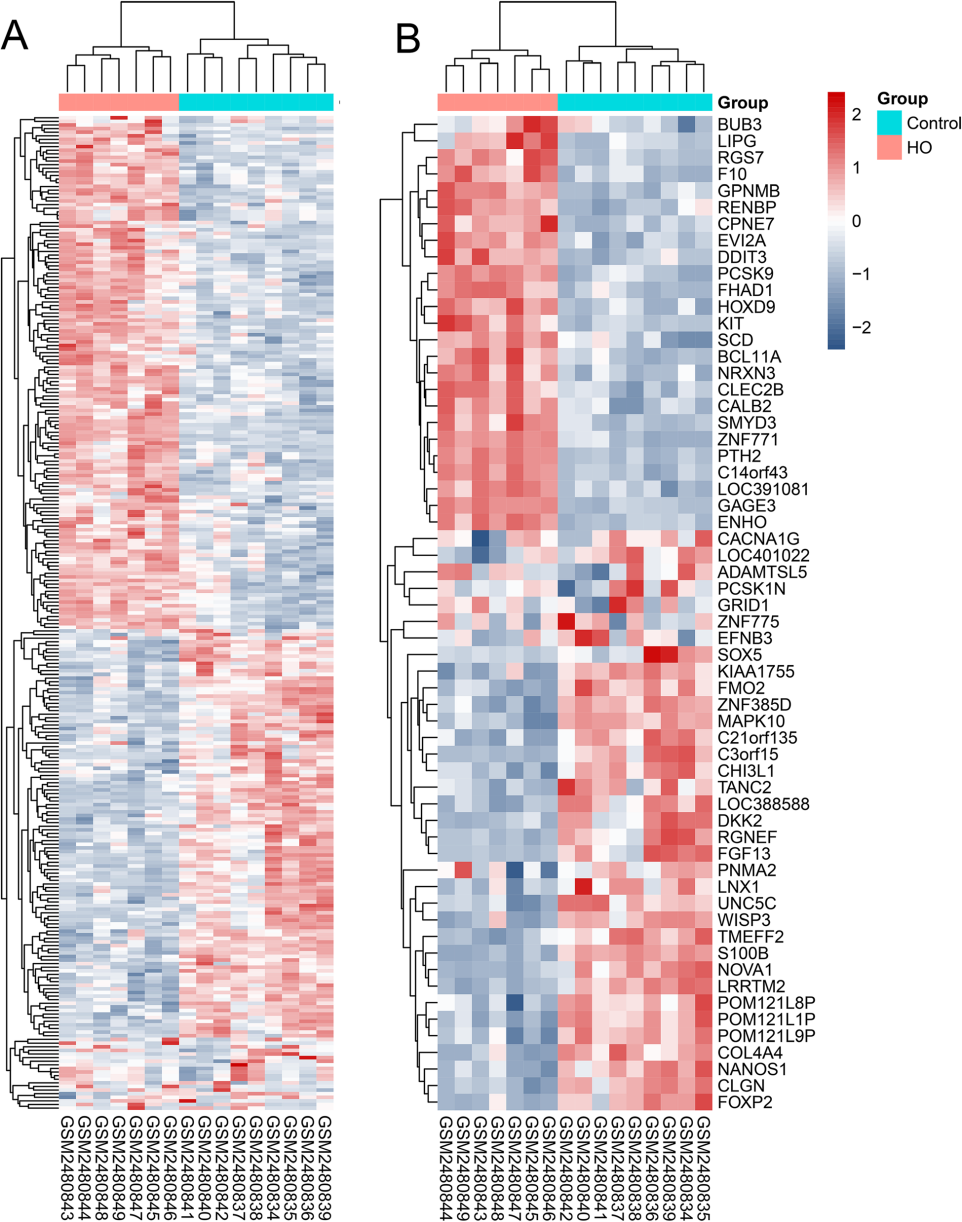
DEGs in expression demonstrated via heatmap. **A** Total DEGs and **B** top 30 DEGs. Red refers to up-regulated expression. Blue refers to down-regulated expression. Max = maximum, min = minimum, BMCs = bone marrow mesenchymal stem cells

A total of 275 DEGs were identified on the basis of the criteria established, including 122 downregulated genes and 153 upregulated genes. The expression levels of the total and the top 30 DEGs are shown in volcano plots and heatmaps (Figs. 3 and 4), indicating that the expression level of DEGs could be used to effectively differentiate the two groups.

### Functional enrichment analysis for DEGs

**Fig. 5:**
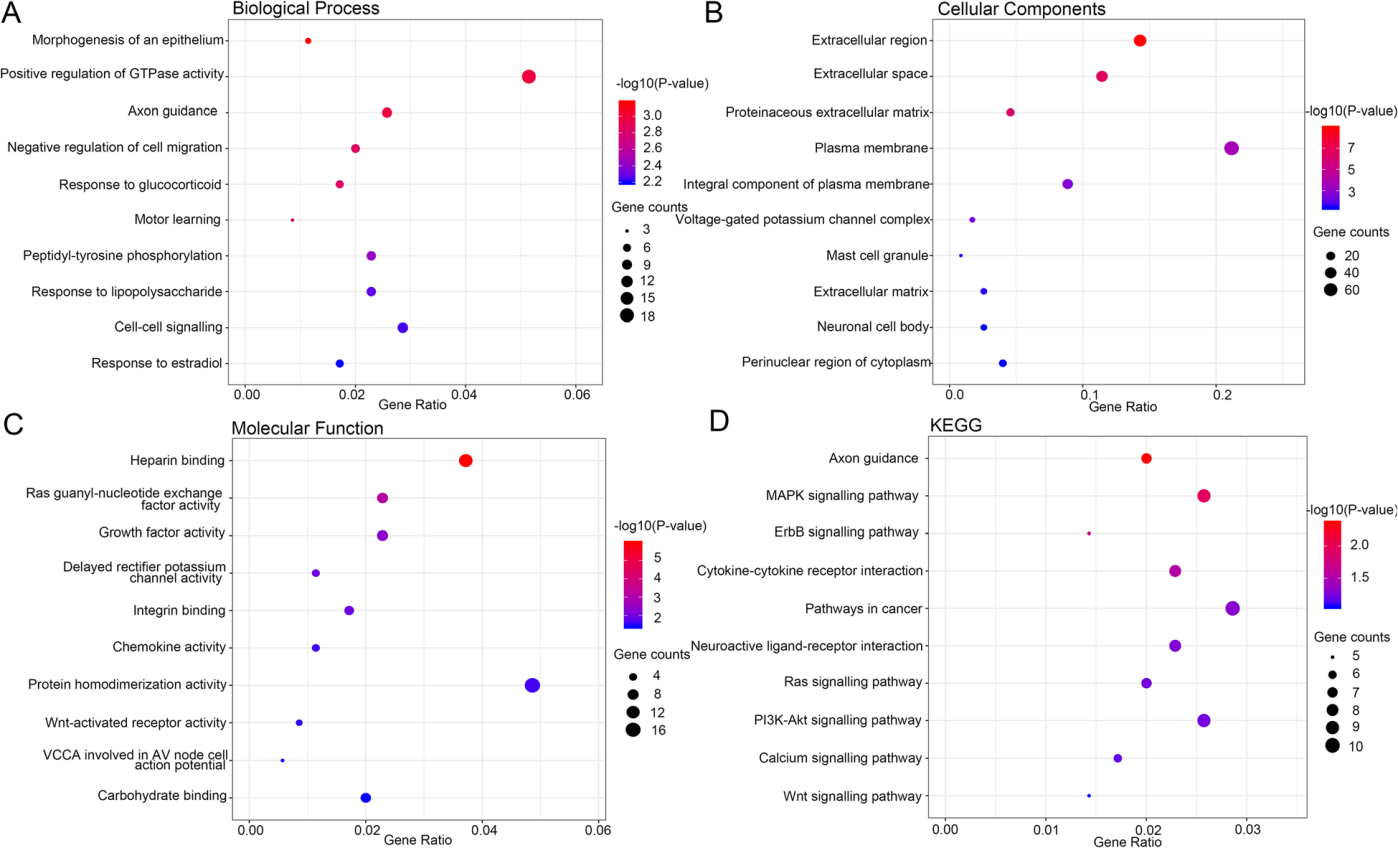
Functional enrichment analysis of DEGs in BMCs from HO tissues demonstrated via bubble plots. **A** Top 10 intensively enriched biological processes in DEGs. **B** Top 10 intensively enriched cell component in DEGs. **C** Top 10 intensively enriched molecular function in DEGs. **D** Top 10 intensively enriched KEGG pathway in DEGs

DAVID was used to perform functional enrichment analyses to explore the biological classification of DEGs and the top 10 items are shown in Fig. 5. In the biological process (BP) ontology, the majority of DEGs, including EFNB3, UNC5C, TMEFF2, PTH2, KIT, FGF13, and WISP3 were intensively enriched in cell signal transmission items, including axon guidance (9 genes), negative regulation of cell migration (7 genes), peptidyl-tyrosine phosphorylation (6 genes), and cell-cell signaling (10 genes). Moreover, KEGG analysis indicated that the majority of DEGs, including EFNB3, UNC5C, FGF13, MAPK10, DDIT3, KIT, COL4A4, and DKK2, were primarily involved in the axon guidance (7 genes), mitogen-activated protein kinase (MAPK) signaling pathway (9 genes), Ras signaling pathway (7 genes), phosphatidylinositol-3-kinase/protein kinase B (PI3K/Akt) signaling pathway (9 genes), and Wnt signaling pathway (5 genes).

### PPI network construction and hub gene selection

**Fig. 6:**
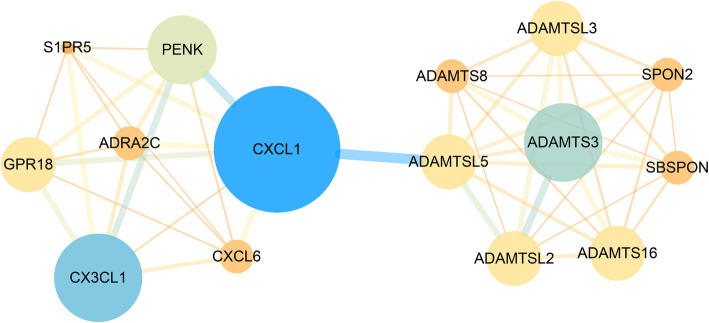
The most significant module was identified with 15 nodes and 50 edges. Small-sized or brightly colored circles and lines indicate a lower value of the combined score

**Fig. 7:**
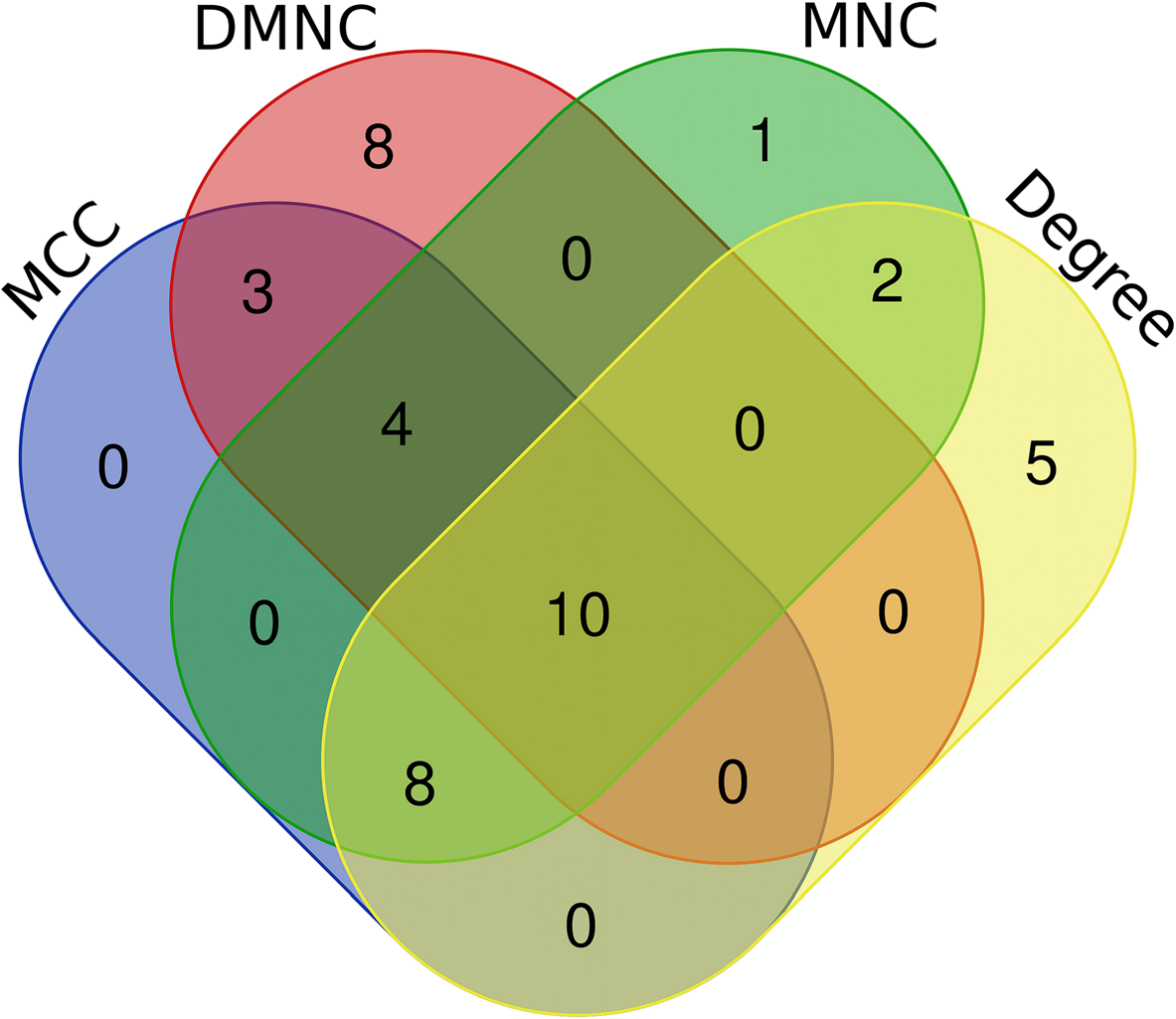
Hub genes identified via a Venn diagram

The PPI network was constructed after the functional relationship and interaction data obtained from STRING were imported into Cytoscape, and the top module was identified (Fig. 6). According to MCC, MNC, DMNC, and Degree, the top 25 genes (10%) were predicted. Venn diagrams were used to select the hub genes, and CX3CL1, CXCL1, ADAMTS3, ADAMTS16, ADAMTSL2, ADAMTSL3, ADAMTSL5, PENK, GPR18, and CALB2, were identified as the hub genes (Fig. 7).

## Discussion

HO refers to a pathological process, involving a variety of etiologies, locations, mechanisms, and cell origins [3]. It has been reported that early diagnosis of HO is difficult due to a lack of evident signs and symptoms and limited treatment [20, 21]. The way to manipulate inflammatory cascades to control HO formation is just beginning to be understood [3]. Therefore, it is urgent to explore the mechanisms of HO formation at the molecular level, to find novel, adequate, and effective treatment methods to alleviate, delay, or even reverse the development of HO.

Under the background of multiple pathological mechanisms, screening possible pathways and targets by means of translational medicine can effectively achieve the research purpose, reduce the research expenditure, and accelerate the time from basic research to clinical application. Whole-genome microarray and bioinformatic analysis facilitate the detection of genetic differences in the development of HO, and provide a better way to explore the pathogenesis and identify novel candidates for early diagnosis and precise treatment. In the current study, 275 DEGs were identified, including 122 downregulated genes and 153 upregulated genes.

After that, functional enrichment analysis was performed, and the relationships and interactions among DEGs were predicted. The majority of DEGs, including KIT, FGF13, EFNB3, UNC5C, TMEFF2, WISP3, and PTH2 were intensively enriched in cell signal transmission items, including axon guidance, negative regulation of cell migration, peptidyl-tyrosine phosphorylation, and cell-cell signaling. Moreover, KEGG analysis indicated that the majority of DEGs, including KIT, DDIT3, FGF13, EFNB3, UNC5C, MAPK10, and DKK2, were primarily involved in axon guidance, the MAPK signaling pathway, the Ras signaling pathway, the PI3K-Akt signaling pathway, and the Wnt signaling pathway. This is consistent with recent research findings that muscle-derived mesenchymal stem cells in soft tissue migrate to the area of trauma and inflammation, differentiate into osteoblasts and form heterotopic bone [22].

The PPI network of the DEGs has been constructed. The results indicate that CX3CL1, CXCL1, ADAMTS3, ADAMTS16, ADAMTSL2, ADAMTSL3, ADAMTSL5, PENK, GPR18, and CALB2 are hub genes.

Chemokines are able to mediate the migration and localization of immunocytes in the process of inflammation, which plays a vital role in manipulating the immune system. According to the conserved cysteine motifs, chemokines in humans are divided into four families (C, CC, CXC, and CX3C). Most chemokines are involved in the differentiation of osteoblasts and/or osteoclasts to varying degrees. CX3CL1, belonging to the CX3C subgroup, is a combination of chemotactic agents and adhesion molecules that has been shown to continuously control reshaping bone matrix by regulating bone remodeling at the cellular level [23, 24]. Current studies have reported that osteoblasts are able to express CX3CL1, and osteoclast progenitors are able to produce CX3C receptor 1 (CX3CR1) [25]. Cytokines originating from inflammation are able to significantly induce the expression of CX3CL1 in osteoblasts. Meanwhile, CX3CR1 was identified as a candidate for screening osteoclasts with inflammatory reactions [26–28]. It was reported that the interaction between membrane-bound CX3CL1 on osteoblasts and CX3CR1 expressed by osteoclast progenitors is able to promote the progress of terminal differentiation of osteoclast progenitors [25]. CX3CR1-deficient mice showed moderate but significantly increased trabecular bone mass, which was mainly due to the decrease in the number of osteoclasts [29]. In vitro experiments suggested that this phenotype can be explained by the decreased ratio of receptor activator of nuclear factor-kappa B ligand/osteoprotegerin (RANKL/OPG), and the defect of spontaneous-formation of osteoclasts from CX3CR1-deficient bone marrow cells [29]. In general, these results indicate that CX3CL1 may be involved in osteoclast-mediated bone loss. CXCL1, belonging to the CXC subgroup, is a kind of growth factor that can send signals through CXC receptor 2 (CXCR2) [30]. CXCR2, a G protein-coupled receptor, is remarkably expressed in osteoclast precursors, though it cannot be predominantly detected in the osteoblast lineage. Cell culture studies have confirmed that recombinant CXCL1 is able to stimulate the migration of osteoclast precursors in a dose-dependent manner [31]. Moreover, CXCL1 has been proven to promote osteoclast formation in vitro [32].

The superfamily of ADAM metallopeptidases with thrombospondin type 1 motifs (ADAMTSs) comprises 19 distinct ADAMTSs, which consist of secreted enzymes and seven ADAMTS-like proteins (ADAMTSLs) without enzymatic activity [33–35]. Most of them are involved in the generation and degradation of extracellular matrix (ECM) molecules, and participate in the formation and remodeling of connective tissue and the occurrence and development of diseases [34, 36]. The ECM of normal cartilage maintains a dynamic balance between generation and degradation, which is in a state of equilibrium. There is a loss of balance between proteases and their inhibitors that degrade the ECM in pathological cartilage. ADAMTS3 has been proven to be involved in the formation of type II fibrous collagen in articular cartilage [37]. ADAMTS16 has not yet been shown to have a specific function in articular cartilage. Current studies have shown that the expression of ADAMTS16 is increased in cartilage and synovium from osteoarthritis patients compared with normal controls [38, 39]. The steady increase in the expression of ADAMTS16 will cause the inhibition of cell proliferation, migration, and adhesion, and a decrease in the expression of matrix metalloproteinase-13 (MMP13) in chondrosarcoma cells [40]. Studies have indicated that ADAMTSLs possess specific extracellular ligands and several of them are ECM-binding proteins that act at the cell-matrix interface [41–43]. It is well known that fibrillin microfibrils are able to bind to ADAMTSLs [44–47]. Therefore, ADAMTSLs can be regarded as matricellular proteins, which are non-structural proteins that express ECM dynamically and have regulatory effects.

PENK, a classically identified opioid gene, was initially shown to be expressed almost exclusively in the mature nervous and neuroendocrine systems. Current studies have revealed that the expression of PENK is selectively increased in mineralized cultures and is essential for the formation and remodeling of bone structure [48]. The expression of PENK in osteoblasts is regulated by bone-targeting hormones, which make a valuable contribution to bone development [49].

The interaction among bone formation and hub genes GPR18 and CALB2 has not yet been reported. GPR18 is a receptor for endocannabinoid N-arachidonyl glycine (NAGly) [50, 51]. GPR18 may be involved in the regulation of the immune system, whose activity is mediated by G proteins that can inhibit adenylyl cyclase [50]. CALB2, a member of the troponin C superfamily, is an intracellular calcium-binding protein that is abundant in auditory neurons and functions as a modulator of neuronal excitability.

## Conclusions

In summary, the current study aims to determine potential biomarkers that might be related to the development of HO and to explore the potential mechanism of HO. In total, 275 DEGs and 8 hub genes were identified, which provided a new promising perspective for HO diagnosis and treatment. The progression of HO may be prevented, alleviated, or even reversed by manipulating these candidate targets in the future. However, the detailed functions of these biomarkers in the pathogenesis of HO need to be studied further.

## Data Availability

All data generated or analyzed during this study are included in published articles.

## Abbreviations

HO: Heterotopic ossification
BMSCs: Bone marrow mesenchymal stem cells
GEO: Gene Expression Omnibus
DEGs: Differentially expressed genes
PPI: Protein–protein interaction
STRING: Search Tool for the Retrieval of Interacting Genes
GO: Gene Ontology
KEGG: Kyoto Encyclopaedia of Genes and Genomes
BP: Biological process
EFNB3: Ephrin B3
UNC5C: Unc-5 netrin receptor C
TMEFF2: Transmembrane protein with EGF like and two follistatin like domains 2
PTH2: Parathyroid hormone 2
KIT: Mast/stem cell growth factor receptor kit
FGF13: Fibroblast growth factor 13
WISP3: WNT1 inducible signaling pathway
MAP K10: Mitogen-activated protein kinase 10
DDIT3: DNA damage inducible transcript 3
COL4A4: Collagen type IV alpha 4 chain
DKK2: Dickkopf WNT signaling pathway inhibitor 2
MAPK: Mitogen-activated protein kinase
PI3K/Akt: Phosphatidylinositol-3-kinase/protein kinase B
CX3CL1: C-X3-C motif chemokine ligand 1
CXCL1: C-X-C motif chemokine ligand 1
ADAMTS: ADAM metallopeptidase with thrombospondin type 1 motif
ADAMTSLs: ADAMTS-like proteins
PENK: Proenkephalin
GPR18: G protein-coupled receptor 18
CALB2: Calbindin 2
FOP: Progressive ossifying fibrous dysplasia
PCA: Principal components analysis
DAVID: Database for Annotation, Visualization and Integrated Discovery
MCODE: Molecular Complex Detection
CX3CR1: CX3C receptor 1
CXCR2: CXC receptor 2
RANKL/OPG: Receptor activator of nuclear factor-kappa B ligand/osteoprotegerin
ECM: Extracellular matrix
MMP13: Matrix metalloproteinase 13
NAGly: N-arachidonyl glycine
